# Elevated Notch1 enhances interleukin-22 production by CD4^+^ T cells via aryl hydrocarbon receptor in patients with lung adenocarcinoma

**DOI:** 10.1042/BSR20181922

**Published:** 2018-12-14

**Authors:** Bo Pang, Cong Hu, Na Xing, Lei Xu, Songling Zhang, Xiaowei Yu

**Affiliations:** 1 Department of Cardiology, The First Hospital of Jilin University, Changchun 130021, Jilin Province, China; 2 Center for Reproductive Medicine, Center for Prenatal Diagnosis, The First Hospital of Jilin University, Changchun 130021, Jilin Province, China; 3 Department of Pediatrics, The First Hospital of Jilin University, Changchun 130021, Jilin Province, China; 4 Department of Thoracic Surgery, China-Japan Union Hospital of Jilin University, Changchun 130000, Jilin Province, China; 5 Department of Oncological Gynecology, The First Hospital of Jilin University, Changchun 130021, Jilin Province, China

**Keywords:** CD4+ T cells, interleukin-22, lung adenocarcinoma, Notch signaling

## Abstract

Notch signaling induced interleukin (IL)-22 secretion by CD4^+^ T cells via retinoid-related orphan nuclear receptor γt (RORγt) or aryl hydrocarbon receptor (AhR). Previous studies have demonstrated that Notch-AhR-IL-22 axis took part in the pathogenesis of chronic viral infection, however, its role in cancer has not been fully elucidated. Thus, the aim of current study was to investigate the involvement of Notch-AhR-IL-22 axis in the pathogenesis of lung adenocarcinoma. A total of 37 late-stage lung adenocarcinoma patients and 17 healthy individuals were enrolled. CD4^+^ T cells were purified from peripheral bloods and bronchoalveolar lavage fluids (BALF), and were stimulated with γ-secretase inhibitor (GSI). mRNA corresponding to Notch receptors and transcriptional factors were measured by real-time PCR. IL-22 concentration was investigated by ELISA. The bioactivity (including cellular proliferation, cell cycle, apoptosis, and invasion) of lung adenocarcinoma cell line A549 was also assessed in response to recombinant IL-22 stimulation *in vitro. Notch1* mRNA expression was significantly elevated in CD4^+^ T cells purified from peripheral bloods and tumor site BALF in lung adenocarcinoma patients. IL-22 expression and *RORγt/AhR* mRNA in BALF was also remarkably increased in tumor site. Inhibition of Notch signaling by GSI did not affect cellular proliferation, but reduced IL-22 production in CD4^+^ T cells from BALF, along with down-regulation of AhR, but not RORγt. Moreover, IL-22 stimulation promoted A549 cells invasion. The current data indicated that elevated Notch1 induced higher IL-22 secretion by CD4^+^ T cells in lung adenocarcinoma patients, and Notch-AhR-IL-22 axis took part in the pathogenesis of lung adenocarcinoma.

## Introduction

Non-small cell lung cancer (NSCLC) accounts for approximately 80% of lung cancer, and mainly comprises adenocarcinoma, squamous cell carcinoma, and large-cell lung cancer [1]. Only a minority of NSCLC patients are detected in early stage with a localized lesion, while most lung cancers are generally diagnosed at advanced, late stages of the disease [2]. Although administrations of molecularly targetted therapies (especially tyrosine kinase inhibitors targetting epidermal growth factor receptor (EGFR)) [3,4] and immune checkpoint inhibitors (including antibodies against programmed death-1 and cytotoxic T-lymphocyte-associated protein 4) [5] have achieved profound therapeutic management of advanced lung cancer [6], it remains the most common cause of cancer-related deaths all over the world [7] due to invasion, migration, and acquired resistance to anti-cancer drugs [8]. More importantly, cancer induces insufficient or dysfunctional immune response, leading to evasion of host immunity [9]. However, the mechanisms which are responsible for hyporesponsiveness are not fully elucidated. Thus, understanding the possible factors will have promising influence in attempting to break immunological tolerance to continuous exposure to cancer antigens.

Interleukin (IL)-22, which belongs to IL-10 cytokine family, is mainly secreted by lymphoid cells, especially CD4^+^ T cells and group 2 innate lymphoid cells (ILCs) [10,11]. IL-22 receptor is restrictedly expressed in non-hematopoietic cells. Thus, signaling through IL-22/IL-22 receptor regulates malignant transformation of epithelial cells and tumor growth mainly by activation of signal transducer and activator of transcription (STAT) 3 [12]. Interestingly, IL-22 is a dual functional cytokine with both proinflammatory and protective activity in context- and dose-dependent manners [13–15]. However, controversy remains as to the expression and function of IL-22 in lung cancer. IL-22 expression was consistently overexpressed in recurrent NSCLC tissue and serum, and promoted cellular proliferation and migration of tumor cells [16]; however, this elevation did not reveal correlation with prognosis of lung cancer patients [17]. Thus, the role of IL-22 in NSCLC requires further investigation.

Notch signaling pathway is a highly conserved signaling system present in most multicellular organisms, and plays a major role in the regulation of cellular development [18]. Activation of Notch signaling drives IL-22 secretion in CD4^+^ T cells via aryl hydrocarbon receptor (AhR), even in the absence of STAT3 phosphorylation [19]. More recent studies also demonstrated that elevated Notch receptors promoted IL-22-producing cells in chronic hepatitis virus infection [20,21]. Thus, we hypothesized that modulation of IL-22-secreting CD4^+^ T cells by Notch signaling pathway is also involved in NSCLC. To test this possibility, we investigated Notch receptors and IL-22 expression, as well as regulatory function of Notch signaling inhibition to IL-22 production *in vitro* in NSCLC patients.

## Materials and methods

### Subjects

The study protocol was approved by the Ethics Committee of The First Hospital of Jilin University and China-Japan Union Hospital of Jilin University. Written informed consent was obtained from each enrolled subject. A total of 37 late-stage (25 in stage III and 12 in stage IV) patients, who were pathologically diagnosed with adenocarcinoma, were enrolled in the current study. All patients were hospitalized in The First Hospital of Jilin University and China-Japan Union Hospital of Jilin University from July 2017 to January 2018. All patients were treatment-naïve, and those who underwent surgery, chemotherapy, or radiotherapy before blood sampling were excluded from the present study. No patients were afflicted by autoimmune disorders, immunocompromised diseases, chronic obstructive pulmonary disease, or pneumonia. All patients were tested for EGFR mutation. Seventeen age- and sex-matched healthy individuals were also enrolled as normal controls (NCs). The clinical characteristics of all enrolled subjects were shown in Table 1.

**Table 1:** Clinical characteristics of enrolled subjects

	Healthy individuals	Lung adenocarcinoma patients
Case (*n*)	17	37
Gender (male/female)	12/5	24/13
Age (years)	48.2 ± 10.9	53.1 ± 8.4
Smoking history (*n*)	4	28
EGFR mutation	N.A.	8

Abbreviation: N.A., not available.

### Isolation of peripheral blood mononuclear cells

Ten milliliters of EDTA-anticoagulant peripheral blood samples were collected from each enrolled subject, and plasma samples were harvested by centrifugation at 3000×***g*** for 10 min. Peripheral blood mononuclear cells (PBMCs) were isolated using Ficoll–Hypaque (Solarbio, Beijing, China) density gradient centrifugation. Approximately 10^7^ of PBMCs could be isolated from 10 ml of peripheral blood.

### Bronchoalveolar lavage fluid preparation

The top of bronchofiberoscope closely wedged into the opening of subsegmental bronchus. Fifty millliters of sterilized saline was rapidly injected through biopsy hole, and the lavage fluids were immediately recovered with 100 mmHg negative pressure. The process was repeated for four times, and the recovery rate was 40–60%. Bronchoalveolar lavage fluid (BALF) was filtrated with sterilized gauze, and was centrifugated at 1200×***g*** for 10 min at 4°C. Supernatants were kept at −70°C, while cellular precipitates were washed twice and harvested for further experiments. Approximately 10^6^ of cells could be isolated from BALF.

### Purification of CD4^+^ T cells

CD4^+^ T cells were purified using human CD4^+^ T cells Isolation Kit (Miltenyi, Bergisch Gladbach, Germany) following manufacturer’s instructions. The purification rate was approximately 20–30%. The purity of enriched CD4^+^ T cells was more than 95% according to flow cytometry determination.

### Cell culture

CD4^+^ T cells were seeded into 24-well plates at a concentration of 10^6^/ml, and were incubated in RPMI 1640 supplemented with 10% of heat-inactivated FBS at 37°C under 5% CO_2_ environment. Cells were stimulated by anti-CD3 antibody (eBioscience, Thermo Fisher, San Diego, CA, U.S.A.; final concentration, 1 μg/ml), with or without Notch signaling inhibitor, γ-secretase inhibitor (GSI) LY-411575 (Adooq, Irvine, CA, U.S.A.; final concentration, 1 μM) for 96 h. Lung adenocarcinoma cell line A549 was used for the study of direct IL-22 modulatory function to NSCLC. A549 cells were confirmed by STR profiling (Procell Life Science & Technology, Wuhan, Hubei Province, China; see Supplementary data). Confirmed A549 cells were cultured in DMEM containing 10% of FBS in the presence or absence of recombinant human IL-22 (Peprotech, Rocky Hill, NJ, U.S.A.; final concentration, 1 μg/ml) for 6 h. Cells and supernatants were harvested for further studies.

### Real-time PCR

Total RNA was purified from cultured cells using RNeasy Mini Kit (Qiagen, Hilden, Germany) following manufacturer’s instructions. First-strand cDNA was synthesized with random hexamers using PrimeScript RT Master Mix (TaKaRa, Dalian, Liaoning Province, China). Real-time PCR was performed using SYBR Premix ExTaq (TaKaRa). Relative gene expression was quantitated by 2^−ΔΔ*C*^_T_ method using Applied Biosystems 7500 System Sequence Detection software (Applied Biosystems, Foster, CA, U.S.A.). The sequences of primers were cited from previous studies [20,21].

### ELISA

Cytokines production was measured using commercial ELISA kit (eBioscience) following manufacturer’s instructions.

### Cellular proliferation assay

Cellular proliferation was measured using Cell Counting Kit-8 (CCK-8; Beyotimes, Wuhan, Hubei Province, China) following manufacturer’s instructions.

### Western blot

A549 cells were lysed on ice for 15 min in 2× SDS loading buffer, and supernatants were collected by centrifugation at 12000×***g*** for 10 min at 4°C. Proteins were separated by SDS/PAGE using Mini-protean Tetra Vertical Electrophoresis Cell System (Bio-Rad, Hercules, CA, U.S.A.), and were eletroblotted on to a PVDF membrane. The membrane was soaked in blocking solution (PBS containing 5% nonfat milk and 0.05% Tween 20) for 1 h, and was incubated overnight in the presence of rabbit anti-p-STAT3 antibody (pY705) antibody or rabbit anti-STAT3 antibody (Abcam, Cambridge, MA, U.S.A.; 1:1000 dilution). The membrane was then incubated with horseradish peroxidase–conjugated polyclonal goat anti-rabbit antibody (Abcam; 1:2000 dilution) for another 2 h. Antibody–antigen complexes were visualized by ECL (Western Blotting Luminol Reagent, Santa Cruz, CA, U.S.A.).

### Flow cytometry

Purified CD4^+^ T cells were stained with anti-CD4-FITC (BD Biosciences, San Jose, CA, U.S.A.) in the dark for 20 min at 4°C. A549 cells were fixed with 70% ice-cold ethanol, and were then stained with propidium iodide (PI, Beyotime) in the presence of RNase (1 mg/ml) for 10 min. Samples were analyzed using FACS Calibur analyzer (BD Biosciences). Acquisitions and analyses were performed using CellQuest Pro software (BD Biosciences).

### Transwell invasion assay

The invasive capacity of A549 cells were measured using Transwell chambers (Corning Costar, Corning, NY, U.S.A.) containing polycarbonate membrane (8 μm pore size), which was covered with 50 μl of Matrigel (BD Biosciences; 1:6 dilution). Cells were suspended in 200 μl serum-free DMEM and were seeded into upper chamber, whereas 600 μl DMEM containing 10% of FBS was added to lower chamber. Cells were cultured for 48 h, and the membrane were stained with 0.1% Crystal Violet for 20 min at room temperature for testing passed cells. The cell numbers were calculated using Olympus BX51 microscope (Olympus, Tokyo, Japan) in five random fields.

### Statistical analysis

All data were analyzed using SPSS 21.0 for Windows (Chicago, IL, U.S.A.). Student’s *t*test was used for comparison between two groups. Paired *t*test was used for comparison between prior to and post stimulation. All tests were two tailed, and *P*-values of less than 0.05 were considered to indicate significant differences.

## Results

### *Notch1* mRNA expression was elevated in CD4^+^ T cells in lung adenocarcinoma

**Figure 1:**
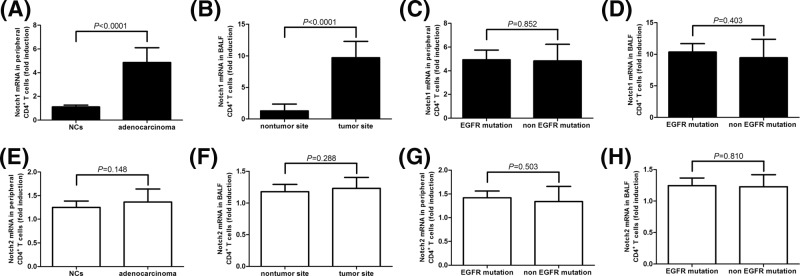
*Notch1* and *Notch2* mRNA expression in peripheral and BALF CD4^+^ T cells from healthy individuals (*n*=17) and lung adenocarcinoma patients (*n*=37) *Notch1* and *Notch2* mRNA expression within 10^5^ of purified CD4^+^ T cells was semi-quantitated by real-time PCR. *Notch1* mRNA was elevated in (**A**) peripheral CD4^+^ T cells from lung adenocarcinoma patients and (**B**) BALF CD4^+^ T cells from tumor site. There were no significant differences in Notch1 mRNA expression in (**C**) peripheral or (**D**) BALF CD4^+^ T cells between EGFR mutation (*n*=8) and non-EGFR mutation (*n*=29) lung adenocarcinoma patients. *Notch2* mRNA expression was comparable in (**E**) peripheral CD4^+^ T cells between NC and lung adenocarcinoma patients and in (**F**) BALF CD4^+^ T cells between nontumor and tumor sites. There were also no remarkable differences in *Notch2* mRNA expression in (**G**) peripheral or (**H**) BALF CD4^+^ T cells between EGFR mutation (*n*=8) and non-EGFR mutation (*n*=29) lung adenocarcinoma patients. Samples for each individual were performed in two independent wells. The columns presented as means, and bars presented as S.D.

CD4^+^ T cells were isolated from all enrolled subjects, and *Notch1/2* mRNA expression within 10^5^ of purified CD4^+^ T cells (either from peripheral blood or BALF) was measured by real-time PCR. mRNA expression corresponding to Notch1 was significantly elevated in peripheral CD4^+^ T cells purified from lung adenocarcinoma patients in comparison with those from NCs (approximately five-fold induction, *P*<0.0001, Figure 1A). This elevation was also found in CD4^+^ T cells isolated from BALF in tumor site rather than in nontumor site (approximately ten-fold induction, *P*<0.0001, Figure 1B). However, there were no remarkable differences in *Notch1* mRNA expression in either peripheral or BALF CD4^+^ T cells between EGFR mutation and non-EGFR mutation lung adenocarcinoma patients (*P*=0.852 and 0.403, respectively, Figure 1C,D). *Notch2* mRNA expression was comparable in peripheral CD4^+^ T cells between NC and lung adenocarcinoma patients (*P*=0.148, Figure 1E) and in BALF CD4^+^ T cells between nontumor and tumor sites (*P*=0.288, Figure 1F). There were also no significant differences in *Notch2* mRNA in either peripheral or BALF CD4^+^ T cells between EGFR mutation and non-EGFR mutation lung adenocarcinoma patients (*P*=0.503 and 0.810, respectively, Figure 1G,H). There were no remarkable difference in Notch receptor expression between stage III and stage IV patients (all *P*>0.05).

### IL-22 expression was elevated in BALF and CD4^+^ T cells from tumor site in lung adenocarcinoma patients

**Figure 2:**
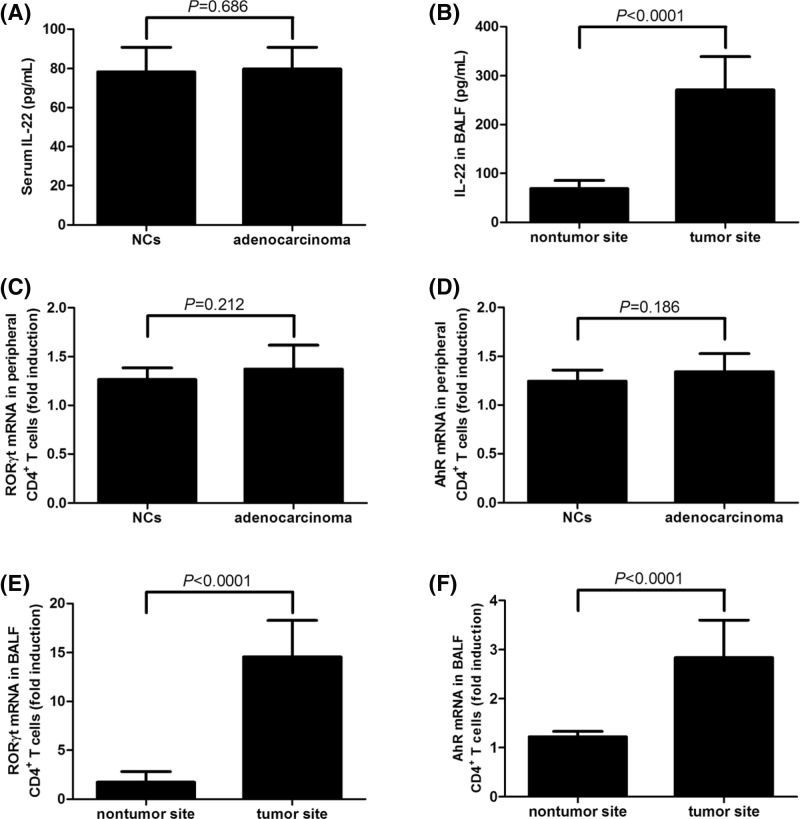
IL-22 and related transcriptional factors expression in peripheral blood and BALF from healthy individuals (*n*=17) and lung adenocarcinoma patients (*n*=37) IL-22 expression in the serum was measured by ELISA. (**A**) Serum IL-22 concentration was comparable between NCs and lung adenocarcinoma patients. (**B**) IL-22 expression in BALF was elevated in tumor site than that in nontumor site. *RORγt* and *AhR* mRNA were semi-quantitated by real-time PCR. (**C**) *RORγt* and (**D**) *AhR* mRNA expression in peripheral CD4^+^ T cells (10^5^ in total number from each subject) was comparable between NCs and lung adenocarcinoma patients. In contrast, both (**E**) *RORγt* and (**F**) *AhR* mRNA was notably increased in BALF CD4^+^ T cells (10^5^ in total number from each subject) from tumor site in comparison with those from nontumor site in lung adenocarcinoma patients. Samples for each individual were performed in two independent wells. The columns presented as means, and bars presented as S.D.

Serum IL-22 concentration was comparable between NCs and lung adenocarcinoma patients (78.27 ± 12.47 compared with 79.78 ± 10.96 pg/ml, *P*=0.686, Figure 2A). In contrast, IL-22 expression in BALF was significantly elevated in tumor site (271.1 ± 67.38 pg/ml) compared within nontumor site (68.67 ± 16.39 pg/ml, *P*<0.0001, Figure 2B) in lung adenocarcinoma patients. Moreover, mRNA expressions corresponding to retinoid-related orphan nuclear receptor γt (RORγt) and AhR were semi-quantitated by real-time PCR in 10^5^ of purified CD4^+^ T cells (either from peripheral bloods or BALF). Neither *RORγt* nor *AhR* mRNA showed remarkable changes in peripheral CD4^+^ T cells between NCs and lung adenocarcinoma patients (*P*=0.212 and 0.186, respectively, Figure 2C,D). However, both RORγt and AhR was notably increased in BALF CD4^+^ T cells from tumor site in comparison with those from nontumor site in lung adenocarcinoma patients (*P*<0.0001, Figure 2E,F). There were also no remarkable differences in IL-22 expression between stage III and stage IV patients (all *P*>0.05).

### Inhibition of Notch signaling pathway suppressed IL-22 production by CD4^+^ T cells from lung adenocarcinoma patients

**Figure 3:**
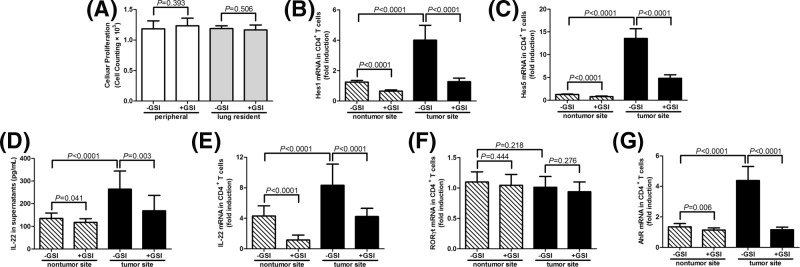
Influence of Notch signaling pathway inhibition on IL-22 secretion by CD4^+^ T cells in lung adenocarcinoma patients CD4^+^ T cells were purified from peripheral bloods or BALF of nontumor and tumor sites, and were cultured with anti-CD3 antibody (1 μg/ml) in the presence or absence of Notch signaling inhibitor, GSI LY-411575 (1 μM) for 96 h. (**A**) A 10^3^ of CD4^+^ T cells from peripheral bloods (*n*=10) or lung resident (*n*=7) were seeded into 96-well plates, and cellular proliferation was measured by CCK-8 in the presence or absence of GSI stimulation. GSI stimulation did not affect CD4^+^ T-cells proliferation. A total of 10^5^ of CD4^+^ T cells from BALF (*n*=23) were then stimulated with GSI for 96 h. IL-22 expression in the supernatants was measured by ELISA, and mRNA expressions corresponding to Hes1, Hes5, IL-22, RORγt, and AhR within CD4^+^ T cells were semi-quantitated by real-time PCR. GSI stimulation down-regulated (**B**) *Hes1* and (**C**) *Hes5* mRNA expression within CD4^+^ T cells from both nontumor and tumor sites. (**D**) GSI stimulation led to the reduction in IL-22 secretion by CD4^+^ T cells purified from both nontumor site and tumor site. (**E**) *IL-22* mRNA expression was also decreased in response to GSI treatment in CD4^+^ T cells from nontumor and tumor sites. (**F**) GSI stimulation did not affect RORγt mRNA expression in CD4^+^ T cells. (**G**) GSI treatment significantly reduced *AhR* mRNA expression in CD4^+^ T cells purified from tumor site, and slightly decreased AhR expression in CD4^+^ T cells purified from nontumor site. Samples for each individual were performed in two independent wells. The columns presented as means, and bars presented as S.D.

We first investigated the impact of Notch signaling inhibition on cell viability; 10^3^ of purified CD4^+^ T cells (ten samples from peripheral bloods and seven samples from BALF of lung adenocarcinoma patients). GSI stimulation did not affect CD4^+^ T cells proliferation from either peripheral bloods (*P*=0.393, Figure 3A) or lung resident (*P*=0.506, Figure 3A). Furthermore, 10^5^ of CD4^+^ T cells, which were from BALF of both nontumor and tumor sites of 23 stage III lung adenocarcinoma patients, were cultured with anti-CD3 antibody in the presence or absence of GSI. Supernatants and cells were harvested 96 h post-stimulation for further analyses. As shown in Figure 3B,C, *Hes1* and *Hes5* mRNA expression were down-regulated in response to GSI stimulation (all *P*<0.0001), indicating the inhibition of Notch signaling pathway by GSI. GSI stimulation led to the reduction in IL-22 secretion by CD4^+^ T cells purified from both nontumor site (135.2 ± 24.02 compared with 117.8 ± 16.40 pg/ml, *P*=0.041, Figure 3D) and tumor site (264.3 ± 79.76 compared with 168.1 ± 68.41 pg/ml, *P*=0.003, Figure 3D). Similarly, *IL-22* mRNA expression was also decreased in response to GSI treatment in CD4^+^ T cells from nontumor and tumor sites (*P*<0.0001, Figure 3E). Furthermore, GSI stimulation did not affect *RORγt* mRNA expression in CD4^+^ T cells (*P*>0.05, Figure 3F). However, GSI treatment significantly reduced *AhR* mRNA expression in CD4^+^ T cells purified from tumor site (*P*<0.0001, Figure 3G), while slightly decreased AhR expression in CD4^+^ T cells purified from nontumor site (*P*=0.006, Figure 3G). Interestingly, IL-22 production and *IL-22/AhR* mRNA was significantly elevated in lung-resident CD4^+^ T cells from tumor site in comparison with those from nontumor site (all *P*<0.0001, Figure 3D,E,G).

### IL-22 stimulation promoted invasion of lung adenocarcinoma cell line A549 cells

**Figure 4:**
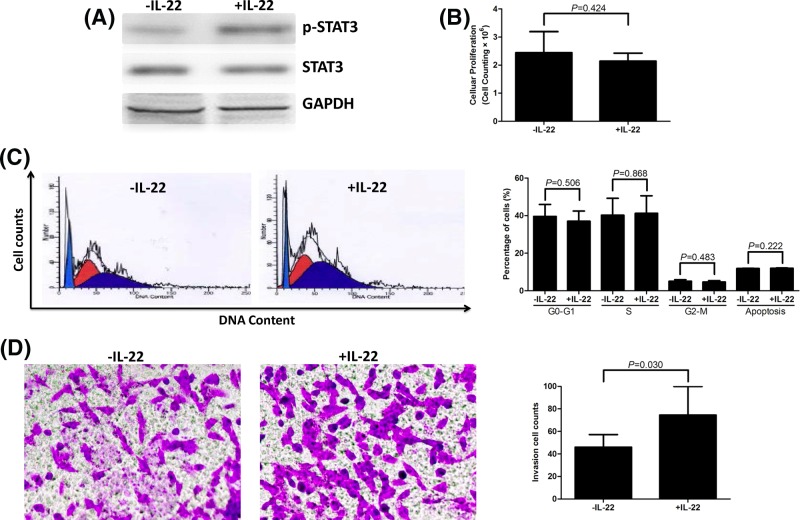
The influence of lung adenocarcinoma cell line A549 bioactivity by IL-22 stimulation A total of 10^6^ of A549 cells were stimulated with or without recombinant IL-22 for 6 h. All cell line experiments were performed as five independent experiments with three replicates in each experiment, and pooled the data from these five experiments for presentation. (**A**) Expression of p-STAT3 and total STAT3 was measured by Western blot, and the representative data were shown. IL-22 stimulation induced the phosphorylation of STAT3, however, total STAT3 level was comparable between prior to and post-IL-22 stimulation. (**B**) Cellular proliferation was measured by CCK-8 method. IL-22 stimulation did not affect cellular proliferation. (**C**) Cell cycle and apoptosis was measured by flow cytometry. Representative PI-stain A549 cells in the presence or absence of IL-22 stimulation were shown. There were no remarkable differences in the percentages of cells in G_0_-G_1_, S, G_2_-M, or in apoptotic phases. (**D**) The invasion of A549 cells was measured by Transwell assay. Representative Crystal Violet-stained A549 cells in the presence or absence of IL-22 stimulation are shown. Cell numbers were calculated in five random fields. Cell numbers, which passed through the membrane, were significantly increased in response to IL-22 treatment. The columns presented as means, and bars presented as S.D.

A total of 10^6^ of A549 cells were stimulated with or without recombinant IL-22 for 6 h, and were harvested for further studies. All cell line experiments were performed as five independent experiments with three replicates in each experiment, and pooled the data from these five experiments for presentation. IL-22 stimulation resulted in STAT3 phosphorylation (Figure 4A), indicating the activation of IL-22 signaling pathway in A549 cells. CCK-8 results showed that IL-22 stimulation did not affect cellular proliferation ((2.15 ± 0.28) × 10^6^ compared with (2.44 ± 0.74) × 10^6^, *P*=0.424, Figure 4B). Flow cytometry was also performed to assess the proportion of A549 cells in different cell cycles and apoptosis. Representative PI-staining A549 cells with or without IL-22 stimulation were shown in Figure 4C. There were no remarkable differences in the percentages of cells in G_0_-G_1_, S, or G_2_-M phases (all *P*>0.05, Figure 4C). In addition, IL-22 also did not lead to notable elevation in apoptotic cells (11.99 ± 0.18 compared with 11.83 ± 0.17%, *P*=0.222, Figure 4C). Moreover, the invasion of A549 cells was investigated by Transwell assay. IL-22 stimulation significantly increased tumor cell invasion (Figure 4D), which presented as elevated cell numbers passing through the membrane in response to IL-22 treatment (74.50 ± 25.35 compared with 46.01 ± 11.19, *P*=0.030, Figure 4D).

## Discussion

In the present study, elevated *Notch1* mRNA expression was found in both peripheral and tumor-resident CD4^+^ T cells in lung adenocarconoma patients. However, increased IL-22 concentration and *RORγt/AhR* mRNA relative level was only found in lung-resident tumor site in lung adenocarconoma patients. *In vitro* IL-22 stimulation promoted the invasion of lung adenocarcinoma cell line A549 cells. Notch signaling inhibition reduced the IL-22 production by CD4^+^ T cells from tumor site, and this process was accompanied by down-regulation of AhR mRNA expression, but not RORγt. The current data indicated the involvement of Notch-AhR-IL-22 axis in the pathogenesis of lung adenocarcinoma.

It has been well accepted that Notch signaling pathway regulated many aspects of cancer biology, especially the cross-talk between different compartments of tumor microenvironment [22] and cancer stem cells in NSCLC [23,24]. Notch signaling played critical roles not only in tumor heterogeneity or tumorigenesis in both NSCLC [25] and small cell lung cancer [26], but also in induction of NSCLC cells resistance by promoting cellular proliferation, triggering migration, and inhibiting apoptosis [24,25,27]. Notch signaling pathway might be potential biomarkers for predicting progression and prognosis of NSCLC patients [25,27]. A more recent study by Li et al. [28] revealed that *Notch2* mRNA was increasingly expressed in both peripheral and lung-resident CD8^+^ T cells, which might contribute to exhausting anti-tumor activity of CD8^+^ T cells in lung adenocarcinoma. However, less studies focussed on Notch receptors expression in CD4^+^ T cells in lung cancer. In the present study, we found that *Notch1* mRNA, but not *Notch2* mRNA, was elevated in both peripheral and lung-resident CD4^+^ T cells in lung adenocarcinoma patients. Notch expression on CD4^+^ cells initiated lung allergic responsiveness by IL-4 induction [29]. Cui et al. [30] have demonstrated that epigenetic status of Notch1 promoter affecting lung-resident CD4^+^ T-cell differentiation in asthmatic rat model. Thus, the current data and previous results indicated an important modulatory activity of Notch1 in driving mature lung-resident CD4^+^ T-cell activation, proliferation, and differentiation [31].

Weidenbusch et al. [32] also revealed an organ-specific gene expression profiles of the Notch-AhR-IL22 axis in humans. Relative high mRNA expression of Notch1, Notch4, AhR, and Δ-like 4 was found in human lung tissue [32]. This was consistent with the current findings in lung-resident CD4^+^ T cells, which showed elevation of Notch1, AhR, and IL-22 in CD4^+^ T cells from lung tumor site. Furthermore, Notch signaling pathway promoted IL-22 production by both CD4^+^ T cells and ILC22 in Concanavalin A-induced hepatitis [19] and chronic viral hepatitis [20,21]. Notch-driving IL-22 secretion was accompanied with elevation of RORγt and AhR in mature gut ILCs [33,34]. However, only AhR was induced in Notch-induced IL-22 production by CD4^+^ T cells [19,21]. In this study, we also revealed down-regulation of IL-22 secretion and *AhR* mRNA expression, but comparable *RORγt* mRNA level, in lung-resident CD4^+^ T cells in response to Notch signaling inhibition in lung adenocarcinoma patients. Increasing evidence supported that IL-22 secretion by CD4^+^ T cells depended on RORγt stimulation and IL-23 production, which were important for Th17 differentiation and IL-6 signaling [35,36]. However, the present and previous studies revealed that Notch signaling pathway was able to induce IL-22 expression from CD4^+^ T cells in absence of RORγt [19], suggesting that Notch-mediated IL-22 expression might be also independent of Th17 differentiation, but dependent on AhR stimulation. However, Notch-AhR-IL-22 axis is a general mechanism for both health and disease. Further experiments were also needed for searching ligands for AhR in the inflammatory regulation by Notch-AhR-IL-22 axis, because there were still increasing numbers of endogenous molecules (e.g. glucosinolates and indole-3-carbinol [37,38]) able to activate AhR.

An elevated IL-22 production was found in BALF from tumor site in lung adenocarcinoma patients, which was consistent with previous results by Tufman et al. [39]. However, IL-22 could be secreted not only by immune cells, but also by tumor cells [40]. Thus, we further showed that IL-22 expression (protein and mRNA) in CD4^+^ T cells from tumor site was elevated than those from nontumor site, indicating that elevated IL-22 expression in BALF might be due to the increased production by CD4^+^ T cells, not lung adenocarcinoma cells. Due to the potential context-dependent protective and promotional effects of IL-22 during tumor formation and progression [11,41], we examined which function of IL-22 might predominate in lung adenocarcinoma cell line by *in vitro* stimulation. IL-22 stimulation led to the phosphorylation of STAT3 in A549 cells, indicating the activation of downstream signaling of IL-22/IL-22 receptor pathway. IL-22 stimulation significantly increased cellular invasion in Transwell culture model. The current *in vitro* results suggested a more potential promotional activity to lung adenocarcinoma. Previous studies revealed that IL-22 directly targetted IL-22 receptor-expressing cells, enhancing proliferation, promoting tumor growth [11], serving antiapoptotic effects on lung tumor cells [17]. However, we found that the elevation of A549 invasion in response to IL-22 was independent of cellular growth promotion since IL-22 did not affect either proliferation or cell cycle/apoptosis of A549 cells. Moreover, STAT3 was an oncogenic signaling pathway, which was a predictor of higher mortality [42] and induced plethora of pro-proliferative and pro-survival factors [43]. Thus, the current data suggested that IL-22 might directly promote lung adenocarcinoma growth by engaging STAT3 signaling. Further *in vivo* experiments are also needed for confirming the results and elucidating the mechanisms.

In conclusion, elevated Notch1 induced higher IL-22 secretion by CD4^+^ T cells in lung adenocarcinoma patients, and Notch-AhR-IL-22 axis took part in the pathogenesis of lung adenocarcinoma.

## Supporting information

**Figure S1:** STR profile of tested A549 cells

**Table S1:** STR profile of tested A549 cells

## Abbreviations

AhR: aryl hydrocarbon receptor
BALF: bronchoalveolar lavage fluid
CCK-8: cell counting kit-8
EGFR: epidermal growth factor receptor
GSI: γ-secretase inhibitor
IL: interleukin
ILC: innate lymphoid cell
NC: normal control
NSCLC: non-small cell lung cancer
PBMC: peripheral blood mononuclear cell
PI: propidium iodide
RORγt: retinoid-related orphan nuclear receptor γt
STAT: signal transducer and activator of transcription
