# Supplementary material for: Elevated Notch1 enhances interleukin-22 production by CD4+ T cells via aryl hydrocarbon receptor in patients with lung adenocarcinoma
Source: Biosci Rep. 2018 Dec 14;38(6):BSR20181922. doi: 10.1042/BSR20181922 (PMC6294615; doi:10.1042/BSR20181922)
Supplement: Supplementary file 1 [file bsr20181922_Supp1.pdf]

Elevated Notch1 enhances interleukin-22 production by $CD4^+$ T cells *via* aryl hydrocarbon receptor in patients with lung adenocarcinoma

Running title: Notch-AhR-IL-22 axis in NSCLC

Bo Pang [1#], Cong Hu [2#], Na Xing [3], Lei Xu [4], Songling Zhang [5]*, Xiaowei Yu [2]*

[#] Bo Pang and Cong Hu contributed equally to this work.

1. Department of Cardiology, The First Hospital of Jilin University, Changchun, Jilin Province, 130021, China

2. Center for Reproductive Medicine, Center for Prenatal Diagnosis, The First Hospital of Jilin University, Changchun, Jilin Province, 130021, China

3. Department of Pediatrics, The First Hospital of Jilin University,, Changchun, Jilin Province, 130021, China

4. Department of Thoracic Surgery, China-Japan Union Hospital of Jilin University, Changchun, Jilin Province, 130000, China.

5. Department of Oncological Gynecology, The First Hospital of Jilin University, Changchun, Jilin Province, 130021, China

* Corresponding author: **Xiaowei Yu** *&* **Songling Zhang**

Center for Reproductive Medicine, Center for Prenatal Diagnosis/Department of Oncological Gynecology, The First Hospital of Jilin University, 71 Xinmin St, Changchun, Jilin Province, 130021, China

E-mail: yuxiaoweipbhc@sina.com (X.Y.) or 13612611@qq.com (S.Z.)

Materials and Methods

STR profiling of A549 cells

Genomic DNA was extracted from 1×10^6 of A549 cells using PureLink Genomic DNA Mini Kit (K182001, Thermo Fisher Scientific, Waltham, MA, USA) following manufacturer's instruction. DNA amplification was performed using PowerPlex 18D System (Promega, Beijing, China), and were analyzed using ABI3500 Genetic Analyzer (Applied Biosystems, Foster, CA, USA).

Results

Confirmation of A549 cells by STR profiling

The STR profile of tested A549 cells were shown in Table S1 and Figure S1, which was identical with the information provided by ATCC (CCL-185).

Table S1. STR profile of tested A549 cells

**Marker**	**Allele 1**	**Allele 2**
D3S1358	16	16
**THO1**	8	9.3
D21S11	29	29
D18S51	14	17
Penta E	7	11
**D5S818**	11	11
**D13S317**	11	11
**D7S820**	8	11
**D16S539**	11	12
**CSF1PO**	10	12
Penta D	9	9
**Amelogenin**	X	Y
**vWA**	14	14
D8S1179	13	14
**TPOX**	8	11
FGA	23	23
D6S1043	11	13
D2S1338	24	24
D12S391	18	18
D19S433	13	13
D1S1656	17	18.3

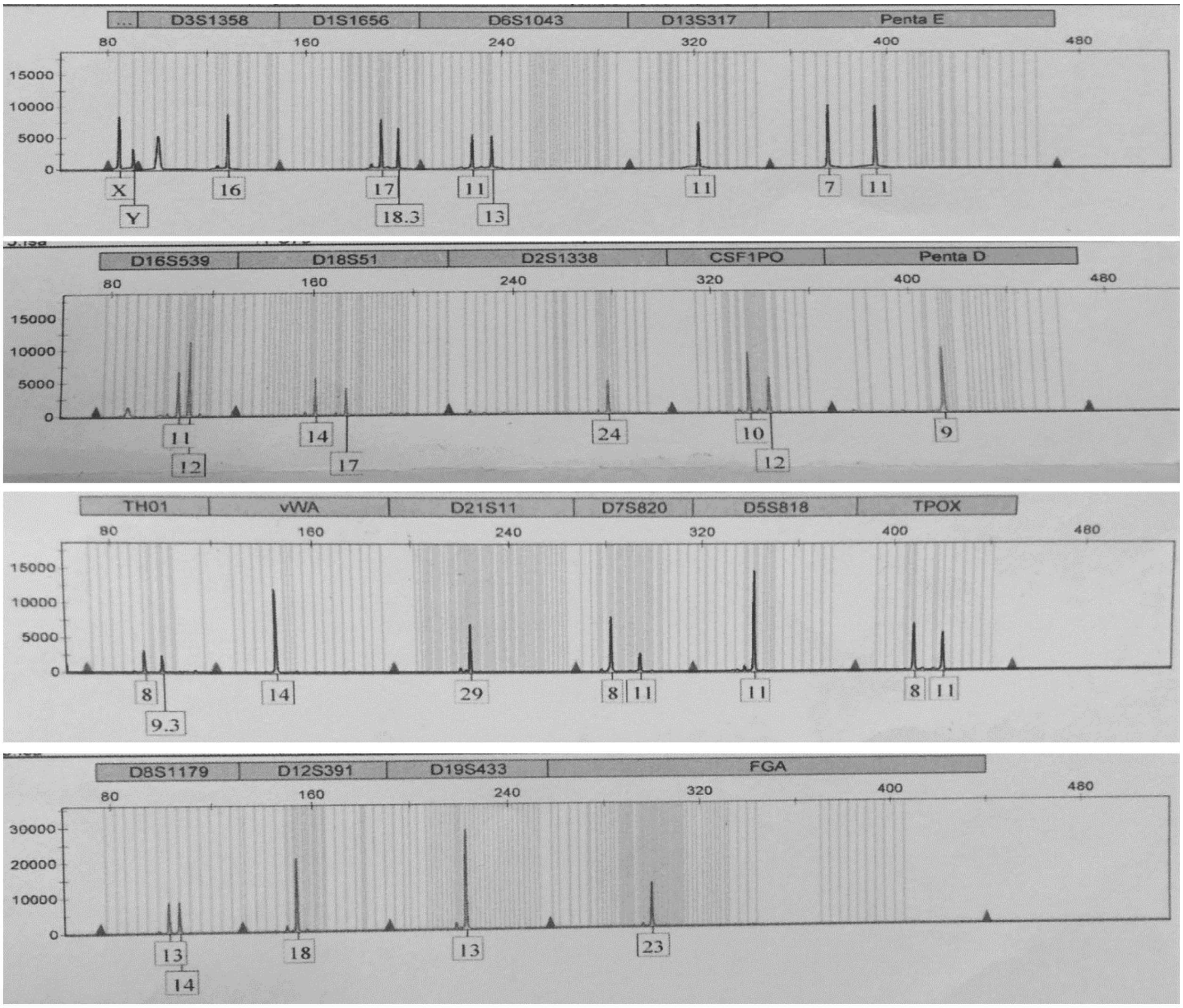

Figure S1. STR profile of tested A549 cells
